# The struggle by *Caenorhabditis elegans* to maintain proteostasis during aging and disease

**DOI:** 10.1186/s13062-016-0161-2

**Published:** 2016-11-03

**Authors:** Elise A. Kikis

**Affiliations:** Biology Department, The University of the South, 735 University Avenue, Sewanee, TN 37383 USA

## Abstract

The presence of only small amounts of misfolded protein is an indication of a healthy proteome. Maintaining proteome health, or more specifically, “proteostasis,” is the purview of the “proteostasis network.” This network must respond to constant fluctuations in the amount of destabilized proteins caused by errors in protein synthesis and exposure to acute proteotoxic conditions. Aging is associated with a gradual increase in damaged and misfolded protein, which places additional stress on the machinery of the proteostasis network. In fact, despite the ability of the proteostasis machinery to readjust its stoichiometry in an attempt to maintain homeostasis, the capacity of cells to buffer against misfolding is strikingly limited. Therefore, subtle changes in the folding environment that occur during aging can significantly impact the health of the proteome. This decline and eventual collapse in proteostasis is most pronounced in individuals with neurodegenerative disorders such as Alzheimer’s Disease, Parkinson’s Disease, and Huntington’s Disease that are caused by the misfolding, aggregation, and toxicity of certain proteins. This review discusses how *C. elegans* models of protein misfolding have contributed to our current understanding of the proteostasis network, its buffering capacity, and its regulation.

**Reviewers:** This article was reviewed by Luigi Bubacco, Patrick Lewis and Xavier Roucou.

## Background

Inherited mutations or polymorphisms, error-prone protein synthesis, environmental stress, and damage accumulated during aging all conspire against cells and organisms by challenging their ability to maintain a healthy proteome. Because cells are under a constantly fluctuating state of proteotoxic stress, there are built-in safeguards − mechanisms by which cells and organisms regulate the rates of protein synthesis, folding, and clearance to minimize protein misfolding and aggregation. The resulting equilibrium in protein metabolism is referred to as protein homeostasis, or simply as “proteostasis” [1].

The study of how proteostasis is regulated has involved molecular chaperone-centric, aging-centric, or disease-centric approaches. *C. elegans* has proven to be an exceedingly useful genetic model system for all three general approaches. Namely, it has been used to study molecular chaperone function, identify genetic pathways that regulate aging, and even express intrinsically aggregation-prone disease-associated proteins as a means to perturb the protein folding environment and investigate how the *C. elegans* proteostasis machinery responds [2, 3].

**Fig. 1:**
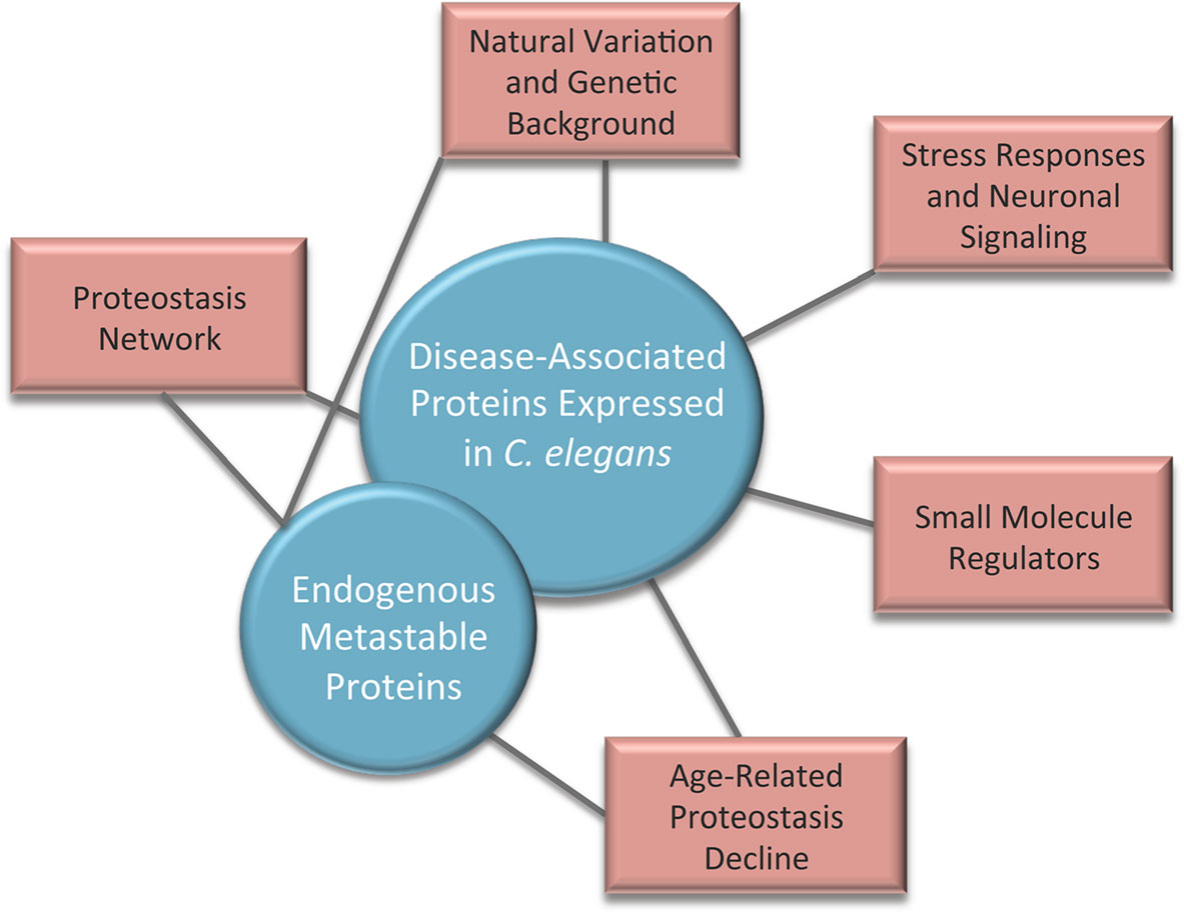
Using *C. elegans* models of protein folding to study the regulation of proteostasis. This review discusses how two different types of folding sensors, disease-associated aggregation-prone proteins and metastable endogenous proteins (shown in *blue*), have been used to uncover the proteostasis network, reveal how natural variation and genetic background modulate the protein folding environment, reveal how proteostasis declines during aging, and identify small molecule regulators of proteostasis (shown in *red*). Black lines connect the two categories of folding sensors to the research areas in which each was employed as a tool

This review focuses on transgenic *C. elegans* in which one of eight different aggregation-prone disease-associated proteins (Htt, ataxin-3, SOD1, Aβ, tau, α-syn, TDP-43 and a polyQ peptide fused to YFP) has been expressed in one of three tissues (body wall muscle, neurons, or intestines). These models (and related models of endogenous misfolded protein) have significantly advanced our understanding of the following:

1. The proteostasis network and how certain cellular factors respond to changes or fluctuations in the protein folding environment.
2. The ways in which genetic background impacts the protein folding environment and consequently the aggregation and toxicity of disease-associated proteins.
3. The declining ability of cells and organisms to regulate proteostasis during aging, perhaps explaining why neurodegenerative disease symptoms do not appear until middle age or later.
4. The role of neurons in controlling proteostasis in a cell non-autonomous manner.
5. The identification of small molecule regulators of proteostasis as potential therapeutic interventions for diseases of protein misfolding (Fig. 1).

### Neurodegenerative disease-associated proteins expressed in *C. elegans*

**Fig. 2:**
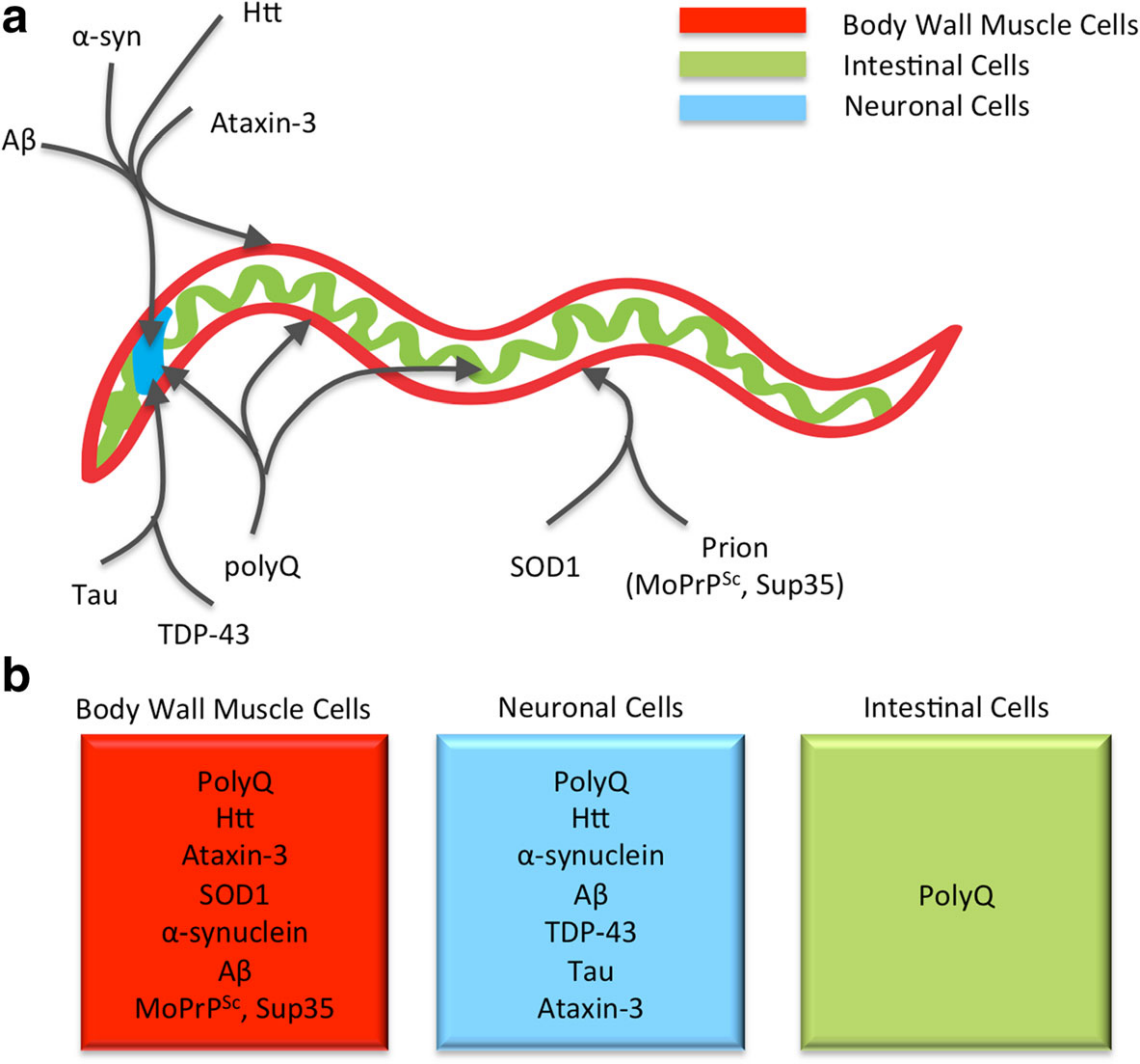
Disease-Associated Proteins Expressed in *C. elegans.* **a** Schematic representation of *C. elegans* showing body wall muscle cells (*red*), neuronal cells (*blue*), and intestinal cells (*green*). Disease associated proteins are indicated and the arrows point to the tissues in which they have been expressed. **b** Each box represents a tissue including body wall muscle cells (*red*), neuronal cells (*blue*), and intestinal cells (*green*). The disease-associated proteins that have been expressed in each tissue are indicated in the corresponding box

Many neurodegenerative diseases are characterized by protein misfolding and aggregation, which seem to be triggers for or causes of neurodegeneration in patients. For example, Alzheimer’s Disease (AD), Parkinson’s Disease (PD), familial amyotrophic lateral sclerosis (FALS)/Lou Gehrig's disease, Huntington’s Disease (HD) and related polyglutamine (polyQ) diseases, such as Spinal and Bulbar Muscular Atrophy (SBMA)/Kennedy’s Disease, Dentatorubral - pallidoluysian atrophy (DRPLA), and spinocerebellar ataxias (SCA) are all characterized by the misfolding and aggregation of proteins and are therefore considered to be conformational disorders [4]. To model protein misfolding in *C. elegans*, aggregation-prone human proteins associated with many of these diseases have been expressed in a variety of *C. elegans* tissues (Fig. 2).

*C. elegans*, with its powerful genetics, relatively simple body plan, and short life cycle is well-suited to model many aspects of protein misfolding, aggregation, and toxicity, to identify genetic modifiers, and to test putative therapeutics. Additionally, many neurodegenerative disease-associated proteins are expressed widely but nonetheless display cell type-specific differences with respect to toxicity. *C. elegans* studies in which human disease-associated proteins are expressed in different *C. elegans* tissues allow such differences to be examined genetically.

Each disease-associated protein expressed in *C. elegans* has its own unique aggregation properties. Even the most closely related proteins, such as those containing a polyQ tract, differ with respect to aggregation propensity and interaction with the protein folding environment. For example, polyQ is much more aggregation-prone on its own than when it is embedded within the context of a disease-associated protein [5, 6]. Furthermore, short fragments of the HD-associated htt protein are less susceptible to protein turnover than longer fragments [7], and the immediate N-terminus of htt has been shown to interact with certain molecular chaperones [8, 9].

#### Models of polyQ protein aggregation and toxicity

PolyQ disorders (HD, MJD, SBMA, DRPLA, and five different spinocerebellar ataxias) are a unique subset of neurodegenerative disorders in that the genetic determinant for each is an expansion of a polyQ-encoding CAG trinucleotide repeat. The polyQ expansion leads to the toxic gain-of-function of a misfolded and aggregation-prone protein. The proteins affected in each polyQ disease are entirely unrelated to each other, outside of the polyQ tract, both in primary sequence and in function. However, the length of the polyQ tract is inversely proportional both to the age of onset of disease symptoms, the severity of symptoms, and in many cases the lifespan of affected patients [10–12].

The first invertebrate model for polyQ protein misfolding, aggregation, and toxicity to take advantage of the power of *C. elegans* genetics was generated in 1999 in the laboratory of Anne Hart [13]. In that model, a polyQ-containing N-terminal fragment of the human htt gene was expressed in *C. elegans* sensory ASH neurons. Animals were generated that had either normal (2 or 23) polyQ repeat lengths or disease-associated polyQ (95 or 150) expansions resulting in ASH neuron degeneration [13].

To further study the phenomenon of polyQ length-dependent aggregation and toxicity, and to consider the hypothesis that the polyQ tract in and of itself is sufficient to cause disease in a manner independent of its normal protein context, the laboratory of Richard Morimoto developed generic polyQ models in which the polyQ tract alone was fused to Yellow Fluorescent Protein (YFP) for visualization in body wall muscle cells [6] or neurons [14]. These studies revealed that polyQ alone fused to YFP (polyQ-YFP) forms aggregates in *C. elegans* in a polyQ length-dependent manner that mimics that which is observed in HD patients. Furthermore, expression of polyQ-YFP, either in body wall muscle cells [6] or neurons [14], led to motility defects, which served as an indication of toxicity. PolyQ-YFP was also expressed in *C. elegans* intestinal cells and used as a sensor for proteotoxic stress that occurs in response to infection [15]. Taken together, these models demonstrated that expanded polyQ tracts are toxic to many different *C. elegans* tissues.

My laboratory focuses on specific polyQ-containing proteins and asks how the sequences flanking the polyQ tract in disease-associated proteins influence aggregation and toxicity. To this end, we have expressed a polyQ-containing C-terminal fragment of the MJD-associated Ataxin-3 protein in body wall muscle cells [5]. The same Ataxin-3 fragment was also expressed in neurons [16]. Likewise, we expressed a polyQ-containing N-terminal fragment of the human htt protein in *C. elegans* body wall muscle cells. In every case, the proteins with expanded polyQ tracts aggregate in and are toxic to the tissues in which they are expressed [5, 6, 14]. However, increased aggregation during aging has been observed for some models [6, 14] but not others [5].

#### Models of Aβ aggregation and toxicity

Alzheimer’s Disease (AD) is the leading cause of dementia worldwide, affecting ~30 % of all individuals over 85 years of age. Its genetics are less straightforward than that of polyQ disorders, as it is polygenic and characterized by the misfolding and aggregation of several different proteins, including the amyloid β (Aβ) peptide and tau. The Aβ peptide is derived from the amyloid precursor protein (APP) and forms amyloid plaques in the brains of AD patients. APP is a single-pass transmembrane protein of which a large domain sits at the extracellular surface. The function of this protein is not well understood, but it may be involved in regulating gene expression in a manner similar to *Notch* [17]. When APP is cleaved sequentially by the proteases α-secretase and γ-secretase at the cell surface, the AD-associated Aβ_1–42_ peptide is not produced. However, certain mutant forms of APP are internalized into vesicles containing β-secretase (BACE1) and γ-secretase that cleave it to produce the disease associated Aβ_1–42_ peptide, which is then secreted and forms extracellular amyloid plaques in a concentration-dependent manner [18, 19].

Consistent with Aβ toxicity being concentration-dependent, Down’s syndrome is characterized by APP overexpression due to trisomy 21 leading to an extra copy of the APP gene. This APP overexpression leads in turn to Aβ deposition in the brains of individuals as young as 30 years of age [20–22]. Likewise, the trinucleotide repeat disorder known as fragile X syndrome leads to an upregulation of APP and Aβ production, leading to impaired neurodevelopment [23]. Finally, a subset of the otherwise healthy population carries the APOE4 allele encoding apolipoprotein isoform 4 that significantly increases the risk of developing AD and is associated with increased Aβ deposition.

Various forms of the disease-associated Aβ peptide have been expressed in *C. elegans* body wall muscle cells and neurons to model aggregation and toxicity. The first of these studies involved either the heat-inducible expression of the N-terminally truncated Aβ_3–42_ peptide in *C. elegans* body wall muscle cells [24] or the expression of that same truncated peptide in neurons [25]. Recently, a full length Aβ_1–42_ peptide was expressed in *C. elegans* body wall muscle cells and shown to be highly toxic, but only to animals grown at elevated temperatures [26]. This suggests that the mechanisms that regulate proteostasis may be able to handle one stress, the Aβ_1–42_ peptide, or heat shock, but not both. This is consistent with the hypothesis that the proteostasis machinery is easily overwhelmed when the misfolded protein load reaches a certain threshold, as elevated temperature may trigger the misfolding of some endogenous proteins. Alternatively, the Aβ_1–42_ peptide may adopt an alternative conformation that is more toxic than that achieved at lower temperatures.

#### Tau, a-syn, SOD1, and TDP-43

Neurofibrillary tangles comprised of a hyperphosphorylated form of the microtubule-associated protein tau, are characteristic not only of AD but also of other tauopathies including an autosomal dominant form of frontotemporal lobar degeneration (FTLD), named FTDL-17 after the affected chromosome. Many mutations in the gene encoding tau on chromosome 17 have been identified and shown to be the underlying genetic determinant of familial FTDL-17 [27, 28]. While there may be a partial loss of function phenotype caused by a failure of mutant tau to interact with microtubules, FTDL-17 appears to be inherited as a dominant disorder likely caused by toxic gain-of-function associated with mutant tau [29]. This toxic gain-of-function hypothesis is supported by the fact that overproduction of tau also leads to FTDL-17. In short, mutant tau is thought to misfold, become hyperphosphorylated, and aggregate in the human brain, leading to characteristic and apparently toxic neurofibrillary tangles [29]. To model tau aggregation and toxicity, the human tau protein was expressed in *C. elegans* neurons and shown to cause neurodegeneration and disrupt neurotransmission [30].

Not all cases of FTDL are familial. In fact, the most common form of FTDL is a heterogenic sporadic disorder known as FTDL-U. Like familial forms of ALS, FTDL-U is characterized by the presence of ubiquitinated aggregates of misfolded proteins. Unlike FTDL-17, the FTDL-U aggregates do not contain any tau. Instead, they have been shown to contain the widely expressed protein known as TDP-43 [31]. TDP-43 contains two RNA recognition motifs and is likely involved in splicing [32]. Interestingly, ubiquitinated TDP-43 aggregates have also been identified in patients with sporadic forms of ALS [31], indicating that these two neurodegenerative diseases have related neuropathologies. To model TDP-43 aggregation and toxicity, has been expressed in *C. elegans* neurons and shown to be neurotoxic [33]. Familial forms of ALS are caused by mutations in Superoxide Dismutase 1 (SOD1) which was also expressed in *C. elegans* body wall muscle cells and shown to aggregate and cause deleterious effects on muscle cell function [34].

Another misfolded and aggregation-prone protein associated with a subset of neurodegenerative disorders is the synaptic protein α-synuclein. α-synuclein aggregates and forms proteinaceous deposits known as Lewy bodies in the brains of PD patients. PD is a progressive neurodegenerative disorder characterized by tremors, rigidity, and loss of dopaminergic neurons in the substantia nigra. The human α-synuclein gene was the first PD gene to be identified [35]. At least three different point mutations in α-synuclein have been described as genetic determinants for rare dominantly inherited familial forms of PD [35–37]. α-synuclein has been expressed in both neurons [38] and body wall muscle cells [39] of *C. elegans* and found to cause neuronal dysfunction and motor loss [38, 39].

α-synuclein has been shown to move from cell to cell in a number of studies including some in which unaffected neurons were grafted to PD-affected brains. The grafted neurons picked up α-synuclein, which in turn seeded aggregation [40–42]. This is reminiscent of the behavior of prion proteins.

#### Prions

Prions are infectious proteins that have the ability to adopt self-propagating conformations. The prion protein (PrP^Sc^) is responsible for transmissible spongiform encephalopathies [43–45]. Like many of the disease-associated proteins described above, PrP^Sc^ forms inclusions of misfolded protein. Interestingly, PrP^Sc^ seeds the misfolding and aggregation of the initially natively folded PrP^C^ [46]. Recent evidence suggests that PrP^Sc^ may not be unique in its infectious nature and that other misfolded and aggregation-prone protein may have prion-like properties [47, 48]. Some of the first evidence that disease-associated aggregates may propagate in a prion-like manner came from the studies with Lewy bodies described above. In addition, injection of Aβ into mice overexpressing APP triggered plaque formation [49]. Finally, HD-associated htt aggregates were shown to be taken up by cultured cells and, once inside, to seed the aggregation of previously soluble htt [50]. Yeast also have endogenous prions, including a region of the Sup35 protein [45]. The prion domain of Sup35 was expressed in *C. elegans* body wall muscle cells where it too displays aggregation and toxicity [51]. The mouse prion protein (MoPrP) was also expressed in *C. elegans* body wall muscle cells yielding a range of phenotypes from completely normal to completely paralyzed [52].

#### Endogenous Metastable Proteins as Folding Sensors

While disease-associated proteins have been used as tools to probe the protein folding environment and elucidate the cellular mechanisms underlying proteostasis, endogenous metastable proteins have also proven to be powerful sensors of the protein folding environment. Specifically, temperature sensitive mutant variants of certain *C. elegans* proteins including paramyosin, myosin, dynamin, ras and perlecan have been used as endogeneous folding sensors. While the temperature sensitive proteins normally fold into their native conformation at permissive temperatures, their folding was shown to be impaired in the presence of expanded polyQ [34, 53] or during other conditions of known or presumptive proteotoxic stress such as aging [54].

### The proteostasis network

Several of these models of protein misfolding in *C. elegans* have been used to elucidate the proteostasis network. The “proteostasis network” refers to the complement of cellular proteins that together are responsible for regulating proteostasis and buffering the folding capacity of cells. Molecular chaperones are obvious candidates for membership in the proteostasis network, as they are able to assist in the folding, disaggregation, and clearance of nascent polypeptide chains and damaged proteins. The protein folding models and sensors described herein are indispensible tools for the identification of factors that comprise the proteostasis network. Because of the availability and ease of use of genome-wide RNAi libraries for gene knockdown in *C. elegans*, many of the first efforts to elucidate the proteins of the proteostasis network involved genome-wide RNAi screens. One study involved a screen for genes that when knocked down resulted in an increase in the aggregation of expanded polyQ-YFP in body wall muscle cells [55]. That study uncovered components of the chaperonin-like CCT molecular chaperone complex as well as genes and proteins that function at different points in the maturation of a protein – as early as transcription and as late as protein degradation. A complementary screen for genes that resulted in a decrease in both polyQ and mutant SOD1 aggregation when knocked down has expanded our list of proteostasis network components to include additional proteins involved in metabolism and RNA processing [56].

Another large-scale genome-wide screen for proteostasis regulators took advantage of a gene expression reporter for the heat shock response (HSR), in which GFP expression was under the control of a heat-inducible promoter. This analysis uncovered genes whose downregulation triggered an HSR in the absence of heat stress and genes whose downregulation suppressed an HSR under conditions when it would normally occur [57]. This study led to the identification of some of the same factors responsible for suppressing polyQ-YFP aggregation, including the CCT chaperonin complex and proteasomal subunits [55, 57], suggesting that these macromolecular machines play particularly important roles in regulating proteostasis.

Taking the slightly different approach of using the temperature sensitive mutants as folding sensors, the Ben-Zvi laboratory identified molecular chaperones that are required for muscle homeostasis in *C. elegans* [58]. Specifically, they uncovered Hsp90 and its co-chaperones Sti-1, Aha1, and Cep23 [58].

Molecular chaperones, consistently being identified in screens for members of the proteostasis network, may comprise the network’s core. Therefore, a targeted study was performed to identify the full complement of molecular chaperones that enhance proteostasis under conditions in which the misfolded protein load was altered by the expression of neurodegenerative disease-associated proteins. All 332 molecular chaperones of *C. elegans* were knocked down by RNAi in animals expressing either Aβ_3–42_ or expanded polyQ-YFP in body wall muscle cells. Yet again, the CCT chaperonin complex was identified along with Hsp90, two Hsp90 co-chaperones (sti-1 and cdc-37), and two DnaJ chaperones (dnj-8 and dnj-12) that likely act as Hsp70 co-chaperones [59].

Interestingly, another DnaJ protein, DNJ-27, was shown to act as a proteostasis regulator based on the fact that its knockdown by RNAi triggered increased aggregation and toxicity of Aβ_3–42_, α-synuclein, and polyQ-YFP [60]. This finding is especially intriguing because DNJ-27 is an ER-resident protein. The Aβ precursor protein, APP, is normally a membrane protein and thus associates with the ER. Likewise, α-synuclein disrupts vesicular trafficking [61], likely feeding back to cause imbalances in ER homeostasis. However, there is no evidence or expectation for polyQ, which is a cytosolic protein, to disrupt the ER. Thus, these data suggest that DNJ-27 levels in the ER affect proteostasis in the cytosol, possibly through signaling or crosstalk.

Other targeted studies have explored whether certain genes or pathways are involved in regulating proteostasis. Namely, the *O*-GlcNac nutrient-dependent post-translational modification, which has been implicated in AD [62, 63], was tested for a role in protein aggregation and toxicity. Specifically, the authors examined the effect of *O*-GlcNac modification in the aggregation and toxicity of pan neuronal tau, Htt-Q150 in ASH neurons, poly-YFP, and Aβ_3–42_ expressed in *C. elegans* body wall muscle cells [64]. These studies revealed that *O*-GlcNac cycling is neuroprotective in *C. elegans* [65] and likely acts by altering rates of protein turnover [66] and by modulating insulin-like signaling [67], an important pathway for *C. elegans* aging.

**Fig. 3:**
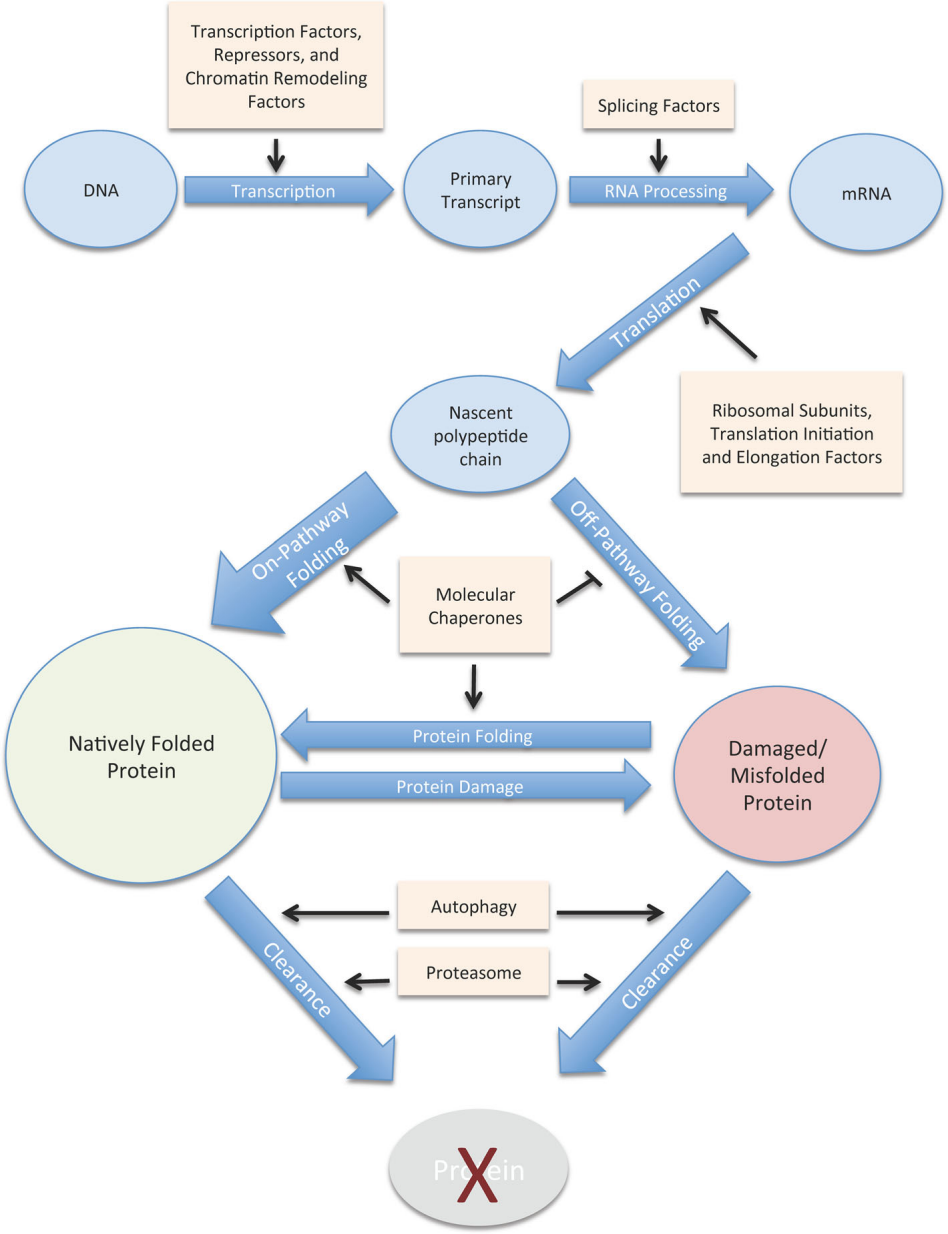
The Proteostasis Network. The pathway for protein synthesis and maturation is shown with specific cellular processes indicated with blue arrows and pathway intermediates indicated in circles. The functional categories of proteins that have been identified as members of the proteostasis network are shown in boxes, with → representing positive regulation and —| representing negative regulation. The thick arrow for on-pathway folding represents the fact that for most proteins under normal non-stress conditions, on-pathway folding predominates over off-pathway folding

Finally, more members of the proteostasis network were identified via proteomics approaches. In one such study, proteins that physically interact with polyQ-YFP were isolated by immuno-precipitation and identified by mass-spectroscopy [68]. Glutamine and asparagine-rich (pqn) proteins were found to have an especially high affinity for binding to polyQ-YFP aggregates. However, one non-pqn protein, CRAM-1, seems to be a primitive molecular chaperone that binds less efficiently than the pqn proteins, but plays a larger role than they in actually blocking aggregate clearance [68]. Together, these findings greatly expand our understanding of the vast scope of proteins and pathways that together regulate proteostasis. In short, the proteostasis network seems to operate at all the points along the pathway for protein synthesis and maturation. Specifically, it includes transcriptional regulators, splicing factors, the protein synthesis machinery including ribosomal subunits, molecular chaperones, and factors involved in protein turnover (Fig. 3).

### Genetic background affects the protein folding environment

**Fig. 4:**
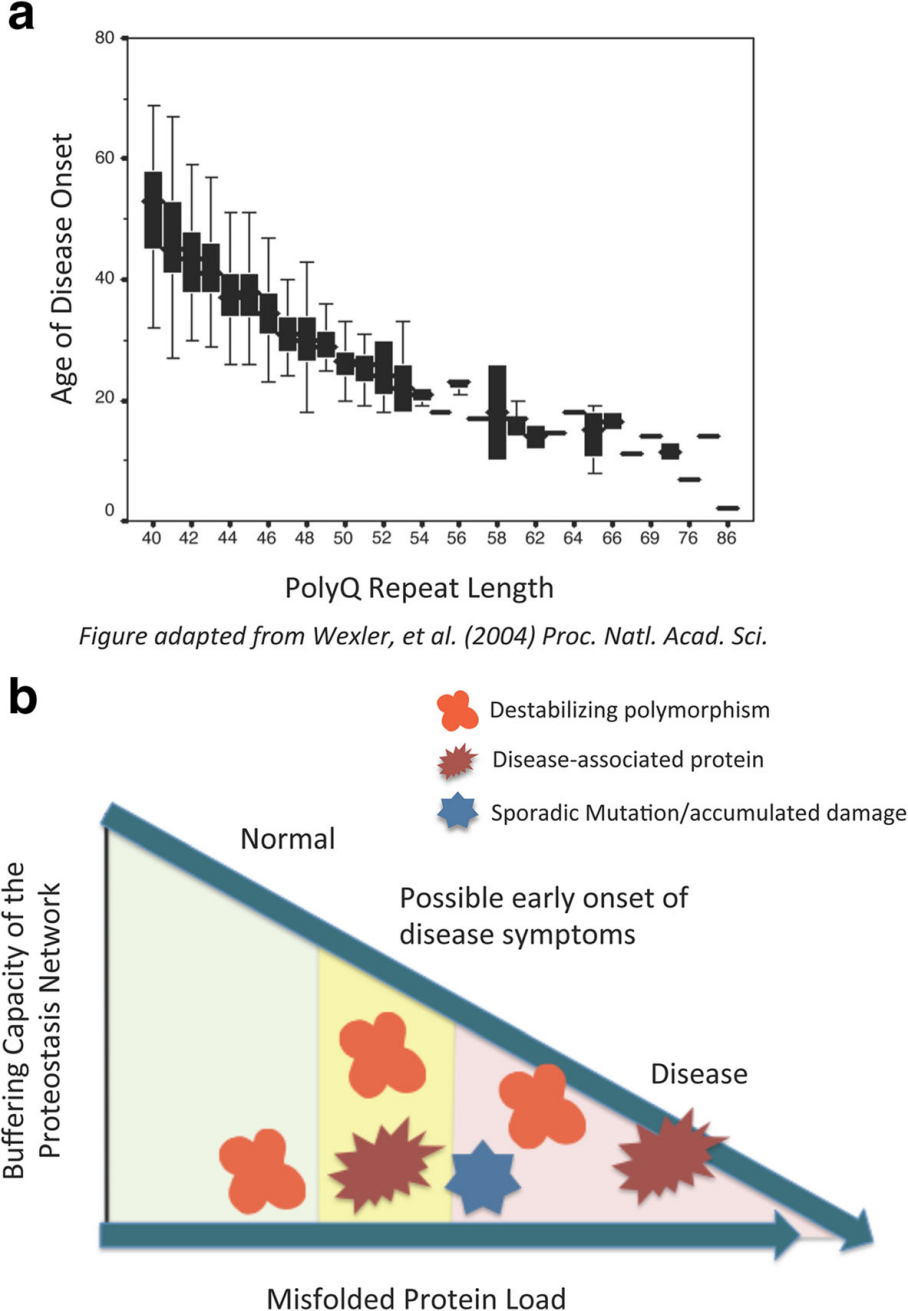
Genetic Background Affects the Protein Folding Environment. **a** Age of Huntington’s Disease onset as a function of polyQ repeat length (adapted from Wexler, *et. al*., (2004) *Proc. Natl., Acad. Sci.*). **b** Schematic representation of the decline in proteostasis buffering capacity as the misfolded protein load increases. Three sources of misfolded protein are considered, including destabilizing polymorphisms in the genetic background (*red*), disease-associate proteins (*brown*), and sporadic mutation to DNA or accumulated damage during aging (*blue*). Symptoms associated with neurodegenerative disease or aging are more likely to be observed when the misfolded protein load is sufficiently high (shaded in *pink*) as compared to normal (*green*) or intermediate levels (*yellow*) of misfolding

The important role that genetic background plays in modulating the folding of the HD-associated htt protein was evident in some of the first studies of HD and its causative genetic anomaly. An inverse correlation between polyQ tract length and age-of-disease-onset was reported at the time that the HD gene was first identified [69]. However, this correlation is not exact, as a certain polyQ length does not perfectly predict the age at which disease symptoms will appear [12]. A more comprehensive study of the role that genetic background plays in modulating age of HD onset was performed using an extensive cohort of HD patients in a remote part of Venezuela [70]. The data showing the large range of age-of-onset, even for the same polyQ lengths within this Venezuelan cohort, is reproduced here (Fig. 4a).

Studies involving some of the *C. elegans* models of protein folding have nicely recapitulated the findings that genetic background influences the protein folding environment and thereby affects the aggregation and toxicity of disease-associated proteins. The first evidence of this in *C. elegans* came from a study using the temperature sensitive mutants to ever so slightly modulate the folding environment in the background of polyQ-YFP expression. A metastable temperature sensitive protein in the background of polyQ-YFP caused polyQ to aggregate more readily than it does in the wild type background [53]. However, the temperature sensitive proteins and polyQ-YFP did not physically interact, meaning that they did not seed the aggregation of each other. Instead, the proteostasis network likely suffers from limited resources, meaning that under conditions that lead to an increased load of misfolded proteins, the network becomes overwhelmed and unable to prevent protein misfolding and aggregation. Furthermore, the expression of ALS-associated mutants of SOD1 in the background of temperature sensitive mutations also resulted in reciprocal misfolding of the temperature sensitive proteins and mutant SOD1 [34, 53]. Together, the findings led to the hypothesis that naturally occurring destabilizing polymorphisms encoded in one’s genetic background may serve to overwhelm the proteostasis machinery, leading to the onset of disease symptoms [2].

To test this, polyQ-YFP expressed in *C. elegans* body wall muscle cells [6] was introgressed into four different wild isolates and used to generate a series of recombinant inbred (RI) lines [71]. The R1350 wild isolate caused the highest amount of polyQ aggregation and the least amount of toxicity [71], fortuitously supporting the hypothesis that the large visible aggregates are cytoprotective rather than toxic [72]. When RI lines were generated by crossing animals carrying the polyQ-YFP transgene in the N2 laboratory background to the DR1350 wild isolate and then inbreeding, a wide range of aggregation propensity was observed, demonstrating that genetic background is a potent modifier of polyQ aggregation and toxicity. This also provides support for the hypothesis that the proteostasis machinery becomes overwhelmed when the number of destabilizing polymorphisms is too high, especially when combined with an aggregation-prone disease-associated protein.

Thus, the ability to achieve proteostasis declines as the load of misfolded protein increases (Fig. 4b). The load of misfolded protein is higher in some genetic backgrounds than others due to the presence of destabilizing polymorphisms. Likewise, the presence of disease-associated aggregation-prone proteins, and damage accumulated over an individual’s lifetime, likely all work together with the genetic background to challenge the proteostasis machinery, leading to an eventual collapse of proteostasis.

### Age-related decline in proteostasis

Damage to DNA and proteins that occurs over the course of aging could be the proverbial final straw that tips the balance of proteostasis away from a healthy proteome and into the realm of disease (Fig. 4b). This is especially true when disease-associated proteins or other metastable proteins are already present. The first evidence that an aging proteome significantly impairs the protein folding environment come from analyzing the temperature sensitive folding reporters during an aging time course. These proteins were shown to misfold at a permissive temperature early in the aging process, indicating that the buffering capacity of the proteostasis network was likely overwhelmed by the combination of a metastable protein and damage accumulated during aging [54].

Even under wild type conditions without the presence of folding sensors or disease-associated proteins in the genetic background, large-scale protein misfolding has been shown to be a hallmark of *C. elegans* aging. Endogenous proteins were separated based on solubility and a measureable increase in the insoluble fraction in older animals was observed [73]. Overexpression of some of the proteins that undergo an age-dependent decline in solubility triggered enhanced aggregation of polyQ-YFP, providing further evidence that an age-dependent decline in proteostasis triggers late age-of-onset of neurodegenerative diseases [73]. Importantly, the mere fact that aggregation of endogenous proteins increases during aging indicates that as damage accumulates to a great enough extent that the proteostasis network becomes overwhelmed even without the additional assault of a disease-associated protein. Thus, a decline in proteostasis may be a cause of normal aging and not just the trigger for the appearance of neurodegenerative disease symptoms.

Large-scale proteomics studies provided yet more evidence that proteostasis decline underpins aging. In one study, 5,000 proteins were profiled during aging, and one third were found to change in abundance, causing loss of normal stoichiometry for important molecular complexes including the ribosome [74]. The upregulated proteins formed insoluble aggregates in an age-dependent manner [74]. In two other studies, the protective heat shock response, a core component of the proteostasis network, was shown to decline measurably during aging [54, 75].

Having shown that the capacity of the proteostasis network to buffer protein misfolding declines precipitously at the transition to *C. elegans* adulthood [54], the Ben-Zvi laboratory asked whether there are certain physiological processes that occur specifically during this developmental transition that result in an increase in proteotoxic stress dramatic enough to overwhelm the proteostasis machinery. To address this, they considered the possibility that the onset of reproduction may be the trigger for proteostasis decline [76]. They found that germ line stem cell arrest rescued proteostasis in a manner dependent on a number of signaling pathways including the heat shock response and the insulin-like signaling pathway [76]. This led to the conclusion that germ line stem cell proliferation likely sends a signal to the somatic cells that suppresses the activity of stress responses thereby leading to an overall decline in somatic proteostasis [76]. This finding is especially noteworthy because it indicates that the buffering capacity of the proteostasis network is so limited that even normal developmental programs cause changes that are too much for the proteostasis machinery to handle.

### Neuronal regulation of protein misfolding and the heat shock response

The heat shock response (HSR) is a transcriptional response to proteotoxic stress that results in the upregulation of the expression of genes that encode molecular chaperones known as heat shock proteins (Hsps). In eukaryotes, the HSR is controlled by the heat shock transcription factor (HSF1), which is activated under environmental conditions that are damaging to proteins, such as a sudden increase in temperature of as little as a few degrees. Under normal conditions when proteins do not experience substantial thermal stress and are thereby in their native conformations, HSF1 is held in an inactive state, outside of the nucleus, via interaction with molecular chaperones. Under conditions of thermal stress, cellular proteins may begin to denature. When this happens, the molecular chaperones release and thereby de-repress HSF1 and bind instead to their preferred partner – misfolded protein. Once de-repressed, HSF1 enters the nucleus, binds as a trimer to the DNA consensus sequence referred to as the heat shock element (HSE) and activates the transcription of Hsps. This results in a negative feedback loop wherein the newly synthesized Hsps not only aid in the folding of damaged cellular protein but also re-repress HSF1 [77–80].

Until relatively recently, most of what we knew about the HSR was based on work utilizing individual cells in tissue culture. To understand how *C. elegans* regulate the HSR on an organismal rather than cellular level, Prahlad *et al.* (2008) examined the HSR in *C. elegans* lacking functional thermosensory AFD neurons. They found that animals whose AFD neurons were genetically ablated with a mutation in the gene *gcy-8* were unable to launch an HSR [81]. Their data provided some of the first evidence that the AFD thermosensory neurons must serve to determine when it is appropriate to launch an HSR and to alert the rest of the animal. The Prahlad laboratory further showed that the organismal HSR in *C. elegans* is dependent on the release of serotonin [82]. Serotonin release not only triggered the HSR, but also suppressed the aggregation of the exogenous polyQ-YFP protein in body wall muscle cells [82]. This is consistent with the previous findings that the *C. elegans* nervous system normally blocks the ability of non-neuronal tissues, including the body wall muscle cells, and intestinal cells to cell autonomously suppress polyQ-YFP or mutant SOD1 aggregation and toxicity [83] and that blocking serotonin reuptake reduces the aggregation of ataxin-3 in *C. elegans* neurons [84].

**Fig. 5:**
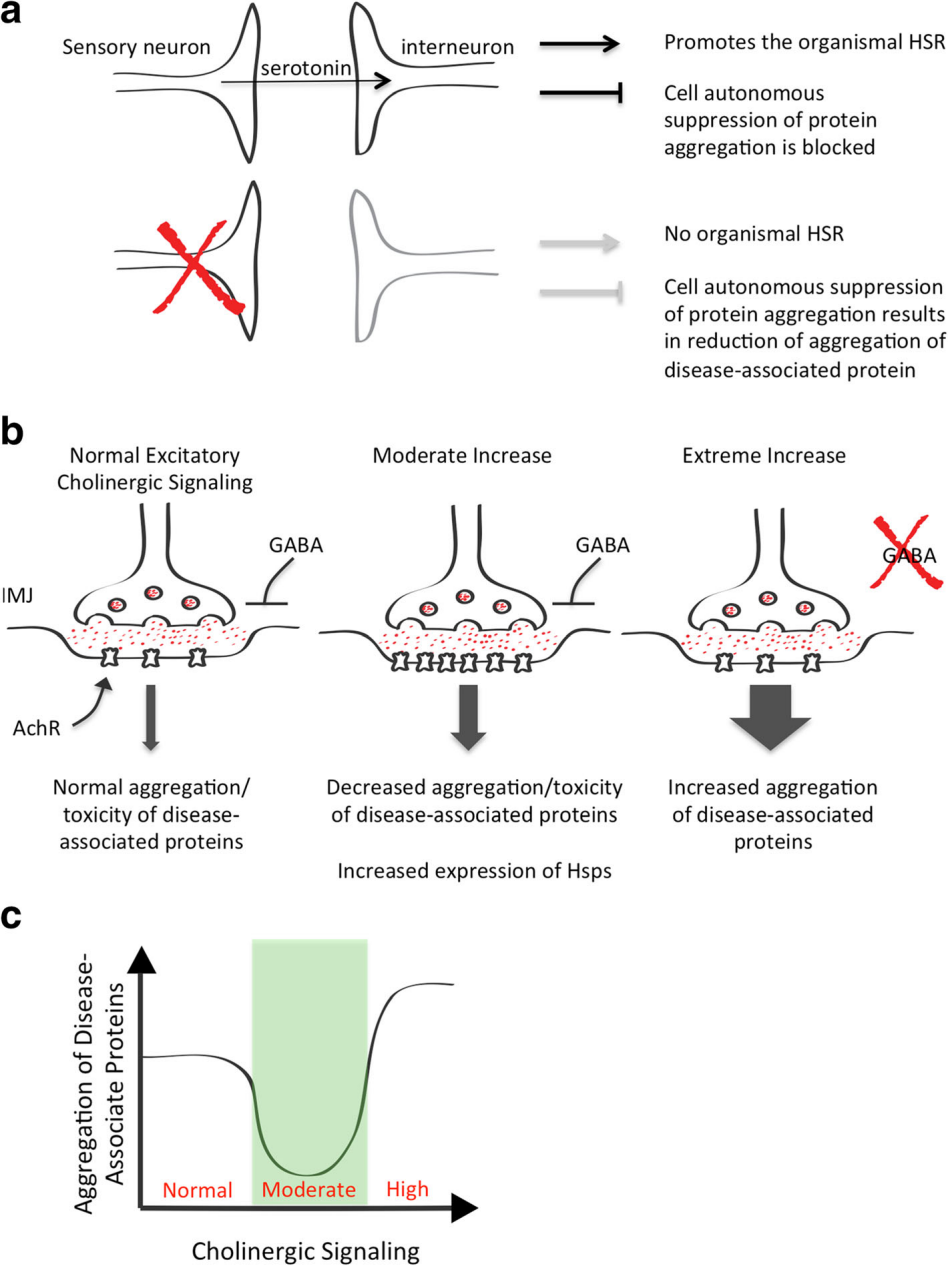
Neuronal Regulation of Protein Misfolding and the Heat Shock Response. **a** Top: Sensory neurons induce the organismal HSR under conditions of acute thermal stress and block the upregulation of Hsps in non-neuronal tissues under conditions of chronic misfolding but normal temperature. Bottom: Genetic ablation of sensory neurons results in an inability to launch an organismal HSR, but de-repression of Hsp expression results in their upregulation in non-neuronal tissues and concomitant suppression of the aggregation of disease-associated proteins. **b** Cholinergic signaling at the neuromuscular junction (NMJ). Red dots represent acetylcholine and the acetylcholine receptor (AchR) is indicated. Left: normal conditions; middle: conditions of moderate increase in acetylcholine signaling to upregulation of the AchR; right: extreme increase in acetylcholine signaling due to lack of the inhibitory neurotransmitter GABA. Downstream effects on aggregation and toxicity of disease-associated proteins are indicated. **c** Schematic graphical representation of the effects of cholinergic signaling on the aggregation of disease associated proteins. The green block represents the narrow window during which acetylcholine signaling is neither too high nor too low to suppress aggregation via the induction of a heat shock response in muscle cells

Taken together, these data suggest that thermosensory neurons act as control centers that induce the HSR under conditions of thermal stress and that block the ability of non-neuronal cells to launch an HSR under normal conditions. This means that cell autonomous heat shock responses cannot occur when individual cells or tissues experience an increase in the aggregation of a disease-associated protein. However, when thermosensory neuron function is ablated, *C. elegans* is unable to launch an organismal HSR, but is able to induce a cell-autonomous response to the presence of disease-associated proteins, thereby reducing protein aggregation (Fig. 5a).

Chemosensory neurons have also been implicated in regulating both the organismal HSR and the toxicity of the Aβ peptide. Specifically, a G-protein coupled receptor (GPCR) that is expressed in chemosensory neurons is required for *C. elegans* to launch an organismal HSR [85]. Additionally, knockdown of this GPCR dubbed *gtr-1* reduced the toxicity of the Aβ peptide [85], reminiscent of the effect of thermosensory neuron disruption on mutant SOD1 or polyQ aggregation [83].

The HSR is only one stress responsive pathway that leads to the upregulation of molecular chaperones. Another important pathway is the insulin/IGF signaling (IIS) cascade that suppresses the activity of the forkhead transcription factor known as DAF-16 in *C. elegans* [86]. DAF-16 activity promotes longevity by inducing the expression of molecular chaperones [87]. A chemosensory protein, NHL-1, acts as a DAF-16 co-factor and is required for stress-resistance in a DAF-16-dependent, but not HSF-1-dependent, manner. Importantly, its knockdown in neurons protects muscle cells from the deleterious effects of the Aβ peptide [88]. Thermosensory neurons regulate the HSR via serotonin release [82], and it is likely that chemosensory neurons work in a similar manner [89].

Cholinergic signaling at the neuromuscular junction (NMJ) also modulates the aggregation and toxicity of disease-associated aggregation-prone proteins expressed in *C. elegans* body wall muscle cells. In one study, cholinergic signaling was significantly upregulated by the complete abrogation of the inhibitory neurotransmitter GABA [90]. In another study cholinergic signaling was only moderately increased due to a mutation that resulted in the upregulation of the acetylcholine receptor at the NMJ [91]. A moderate increase in cholinergic signaling suppressed the aggregation/toxicity of the disease-associated proteins polyQ-YFP and mutant SOD1, and suppressed the misfolding of the temperature-sensitive folding sensors [91] (Fig. 5b). In contrast, extreme upregulation of cholinergic signaling increased the aggregation of polyQ-YFP [90], indicating that the ability of *C. elegans* to regulate proteostasis is highly sensitive to relatively slight changes in cholinergic signaling at the NJM (Fig. 5b). Thus, there seems to be a narrow window of cholinergic signaling levels that is able to mitigate the deleterious effects of disease-associated proteins [91] (Fig. 5c). Cholinergic signaling at the NMJ and signaling from the serotonergic sensory neurons likely work together to balance proteostasis in non-neuronal tissues in response to thermal stress and changes to the load of misfolded proteins due to aging and disease.

### Small molecule regulators of proteostasis

We have learned a great deal using *C. elegans* as a model system to study the cellular and molecular biology underlying conformational diseases and the maintenance of proteostasis. One important finding is that while the proteostasis network is controlled in many ways, including by neurons, its capacity to buffer against protein misfolding is limited. This means that subtle changes in the folding environment that occur during aging can significantly impact the health of the proteome. The ability to pharmacologically boost the network’s buffering capacity has the potential to serve aging members of our society, especially those at risk for neurodegenerative disease. Several screens for small molecule regulators of proteostasis have led to the identification of candidate molecules.

While upregulation of molecular chaperones seems to be an obvious strategy for mitigating the toxic effects of misfolded aggregation-prone disease-associated proteins by helping them to attain their native conformations, there is some evidence that inhibiting molecular chaperones may actually switch the balance from folding to degradation, thereby promoting the clearance of toxic proteins [92]. This suggests that any sort of treatment that tips this balance, including, perhaps, molecular chaperone downregulation and direct activation of clearance pathways, may likewise be effective in ameliorating the toxic effects of misfolded aggregation-prone protein.

In addition to directly targeting molecular chaperones for pharmacological activation or inhibition, the heat shock response has been targeted to increase molecular chaperone levels in the absence of thermal stress. Calamini et al. (2012) screened a library of 900,000 small molecules for their ability to induce an HSR and reduce the aggregation of polyQ expanded human htt protein in cells in tissue culture [93]. The small molecules identified were then tested for their ability to suppress polyQ-YFP aggregation in *C. elegans* body wall muscle as evidence of the molecules working in the context of an organism. Three small molecules were identified that met all of these criteria [93].

At face value, small molecules that disrupt proteostasis by causing protein misfolding and damage may be expected to induce an HSR. However, stress-independent activation of the HSR via the direct activation of HSF1 suppresses the aggregation/toxicity of disease-associated proteins [94]. Specifically, when HSF1 was activated, molecular chaperone levels rose sufficiently for a suppression of polyQ-YFP aggregation in *C. elegans* body wall muscle cells [94].

Because the transcription factor DAF-16 also controls molecular chaperone expression, pharmacological activation of DAF-16 directly or indirectly via the suppression of the IIS pathway may be a viable method to increase the buffering capacity of the proteostasis network and simultaneously to increase lifespan and reduce the aggregation and toxicity of disease-associated proteins. To identify such small molecules, the Cohen laboratory screened a family of IIS inhibitors [95] and found one that suppressed Aβ_3–42_ and polyQ-YFP aggregation and toxicity in *C. elegans* body wall muscle cells [96]. Interestingly, this inhibitor had no effect on longevity [96].

The laboratory of Stephen Pak performed two screens for small molecules that suppressed the aggregation and reduced the accumulation of the mutant form of α1-antitrypsin (ATZ) that is the genetic determinant for the disease α1-antitrypsin deficiency. In one study, they developed a high-content drug screen that revealed 33 such small molecules [97]. In a follow-up screen, they performed genome-wide RNAi to identify genes that when knocked down either increased or decreased the accumulation of the ATZ variant of α1-antitrypsin in *C. elegans*. They then identified four FDA approved drugs that either enhanced or suppressed the activity of the genetic modifiers and consequently suppressed the accumulation and aggregation of α1-antitrypsin ATZ mutant [98]. Together, these data support the therapeutic potential of small molecule proteostasis regulators to suppress the toxicity of proteins associated with conformational disease and aging.

## Conclusions

*C. elegans* models of protein misfolding have contributed greatly to our current understanding of the proteostasis network, its buffering capacity, and its regulation. Fourteen different models of protein misfolding have been described, including nine different neurodegenerative disease-associated proteins, each expressed in up to three possible tissue types. One of the most striking findings to come from studies of these models is just how limited cells and organisms are in their ability to buffer protein misfolding. Neurons play an integral role in regulating the organismal HSR and seem to contribute to the limited buffering capacity − at least when the misfolded protein load increases in the absence of a triggering environmental stress such as heat shock. Finding ways to combat this inherent limitation in the buffering capacity of the proteostasis network is crucial for the treatment of patients suffering from protein folding disorders. To this end, many small molecules have been identified for the purpose of pharmacologically enhancing the ability of cells and organisms to handle the expression of chronically misfolded disease-associated proteins. An important next step will be verifying the efficacy of these molecules in mammalian models and human patients. Such validation is especially important because the *C. elegans* models described here are designed to recapitulate only very specific and limited aspects of disease, and therefore cannot be treated as “disease models” *per se*.

## Reviewers’ comments

*I would like to thank all of the reviewers for taking the time to carefully review this manuscript and provide thoughtful comments and suggestions. I have carefully considered all of the suggestions and have addressed each of them in the text as described below.*

### Reviewer report 1

Luigi Bubacco, University of Padua

## Reviewer comments

### Recommendations

This is an interesting and comprehensive review centered on the role that the model organism *Caenorhabditis elegans* had in the investigation of protein misfolding and in the unraveling of the processes that govern proteostasis. This is a balanced and sound analysis of the exiting literature, nicely written and I endorse it for publication. Few considerations are due to improve readability: Line 68: The author may consider the possibility to provide to the readers the rationale to embark in the expression of the misfolding proteins in the different type of tissues. It may be of interest to comment on the different aggregation properties of the thirteen different paradigms of protein misfolding described in the review and how these properties impact on the proteostasis.

Author’s response: *Thank you for your kind words and helpful suggestions. I have addressed your comments regarding “rationale” and “aggregation properties” in the text with the addition of two paragraphs at the end of the section titled “*Neurodegenerative disease-associated proteins.”

### Reviewer report 2

Patrick Lewis, University of Reading

## Reviewer comments

### Summary

The manuscript from Kikis reviews the biology of proteostasis relating to the experimental organism *C. elegans* in health and disease. This is an important area of research, as using *C. elegans* as a model system allows whole organism investigations into proteostasis that are difficult to carry out in other organisms. These studies provide insights into the fundamental biology of proteostasis, but also have important implications for a number of human diseases. The review focuses on a range of neurodegenerative diseases associated with protein aggregation and accumulation for which worm models have been generated, and is structured around noting what models have been reported in for a range of disorders, before moving on to summarize how different aspects of the proteostasis machinery have been investigated in relation to these protein aggregates.

### Recommendations

The background on the genetics and clinical presentation of some of the proteins under consideration, for example huntingtin, is excellent - however more detail is needed for a number of the disorders presented. For example, when discussing Alzheimer’s there is no reference to the monogenic forms of the disease. Mutations in APP, in particular, are critical for understanding the role of abeta species in AD - and it would also be useful to note the impact of gene dosage of APP on the risk of dementia, both in terms of gene duplications and chromosomal copy number variations in the case of trisomy 21. This is of particular importance when considering the genetic evidence for protein burden per se being a key issue in disorders of proteostasis.

Author’s response: *The section on AD has been significantly fleshed out in the revised manuscript. In particular, APP mutations are now discussed along with the roles of α, β, and Y secretases in APP processing and the formation of disease-associated forms of Aβ. In addition, the involvement of Aβ in Down’s Syndrome and fragile X syndrome are addressed in the revised manuscript.*

Likewise, the descriptions of tau, alpha synuclein, tdp43 and sod1 are cursory. At a minimum there should be a brief description of the genetic evidence linking these proteins to disease - in the case of tau, mutations in MAPT causing frontal lobar degeneration, in the case of synuclein mutations and gene duplications/triplications (linking in to protein level burden) causing familial Parkinson’s and dementia with lewy bodies.

Author’s response: *The section on tau has been expanded to include a discussion of MAPT mutants causing familial forms of frontotemporal lobar degeneration as suggested. In addition, the sections on TDP-43, alpha-synuclein, and prions have been significantly expanded with respect to mechanisms of action and disease-associated mutations.*

The section of prion disorders is also very brief, and misses several references including citations for models examining PrP expression in c elegans.

Author’s response: *Thank you for pointing this out. The omission has been rectified with the* C. elegans *model for mouse PrP now discussed in the text and added to Fig.* *2* *.*

It would also be of great interest to link prion behavior across all of these proteins, an area of considerable research at present.

Author’s response: *The section on prions now includes a discussion of recent data suggesting that all the aggregation-prone disease-associated proteins discussed in this review article may have prion-like properties.*

There is a detailed description of reports relating to different proteostasis systems in *C. elegans*, with an emphasis on protein chaperone responses. It would be useful to provide a brief overview of the different systems regulating protein production and degradation, perhaps as a figure, which could be used to synthesize how these systems work in concert.

Author’s comments: *To address this suggestion, a new figure (Fig.* *3* *) was added that shows the pathway of protein synthesis and maturation and the functional categories of proteostasis network components that act at each step.*

One aspect of the manuscript that is in need of attention is highlighting the drawbacks of using *C. elegans* in the context of human neurological disorders. It is notable that many of the proteins that are examined in the reported models do not have paralogs in the *C. elegans* genome, and while this provides an excellent opportunity to dissect out certain aspects of the disease process by taking a reductionist approach it does have drawbacks in terms of extrapolating to the human disease situation.

Author’s comments: *To address this suggestion, I now conclude the paper with a sentence clarifying that the* C. elegans *models described here are not meant to recapitulate all aspects of disease and thus “cannot be treated as ‘disease models’ per se.”*

### Reviewer report 3

Xavier Roucou, Université de Sherbrooke

## Reviewer comments

### Summary

*C. elegans* models of protein misfolding have contributed greatly to our current understanding of the proteostasis network. This is a timely review in the field and it will be very helpful for the scientific community in general. The proteostasis community may also find this review useful. The review is easy to read.

### Recommendations

Major recommendations 1. An entire section (pages 9–12) in the review reports many studies that used different strategies to identify key members of the proteostasis network (proteins and pathways that together regulate proteostasis). The term "proteostasis network" appears several times in the review. Yet, besides some chaperones that are mentioned in the text, this network remains mysterious. I suggest to include a figure of the proteostasis network that includes the key players. Obviously, the idea is not to write a list of all players that were identified in C. elegans, but to give an overview of what kind of regulators and pathways are involved.

Author’s comments: *Thank you for this suggestion. It is similar to one also made by reviewer #2. As mentioned above, I added a new figure (Fig.* *3* *) to the revised manuscript. It shows the pathway of protein synthesis and maturation and the functional categories of proteostasis network components that act at each step.*

2. In the abstract, the author writes that "Additional demand is placed on the proteostasis machinery during aging by a gradual increase in misfolded protein that results from accumulated molecular damage triggered by reactive oxygen species or other mutagens". Yet, this issue is not discussed at all in the review. I recommend to either elaborate on that topic in the review, or to remove this sentence from the abstract.

Author’s comments: *The sentence to which the reviewer is referring originally stated “Additional demand is placed on the proteostasis machinery during aging by a gradual increase in damaged and misfolded protein that results from accumulated molecular damage triggered by reactive oxygen species or other mutagens.” However, as the reviewer noted, I do not discuss reactive oxygen species or mutagens. As those topics are outside the scope of this article, I revised the sentence to say, “Aging is associated with a gradual increase in damaged and misfolded protein, which places additional stress on the machinery of the proteostasis network.”*

### Minor recommendations

Page 11: alpha-synuclein is not a lysosomal protein. It enters into the lysosome, where the protein is degraded. But it is not a normal resident of the lysosome. Page 18: give the name of the GPCR in the text.

Author’s comments: *These errors and omissions have been corrected in the revised manuscript.*

## Abbreviations

AD: Alzheimer’s disease
ALS: Amyotrophic lateral sclerosis
APP: Amyloid precursor protein
Aβ: Amyloid beta
ER: Endoplasmic reticulum
GPCR: G-protein coupled receptor
HD: Huntington’s disease
HSF1: Heat shock factor 1
Hsp: Heat shock protein
HSR: Heat shock response
Htt: Huntingtin protein
NMJ: Neuromuscular junction
PD: Parkinson’s disease
polyQ: Polyglutamine
PrP: Prion protein
YFP: Yellow fluorescent protein
